# Zebrafish Models of *LAMA2*-Related Congenital Muscular Dystrophy (MDC1A)

**DOI:** 10.3389/fnmol.2020.00122

**Published:** 2020-07-09

**Authors:** Lacramioara Fabian, James J. Dowling

**Affiliations:** ^1^Program for Genetics and Genome Biology, Hospital for Sick Children, Toronto, ON, Canada; ^2^Division of Neurology, Hospital for Sick Children, Toronto, ON, Canada; ^3^Departments of Pediatrics and Molecular Genetics, University of Toronto, Toronto, ON, Canada

**Keywords:** laminin, LAMA2 gene, muscular dystrophy, zebrafish model, MDC1A

## Abstract

LAMA2-related congenital muscular dystrophy (CMD; LAMA2-MD), also referred to as merosin deficient CMD (MDC1A), is a severe neonatal onset muscle disease caused by recessive mutations in the LAMA2 gene. LAMA2 encodes laminin α2, a subunit of the extracellular matrix (ECM) oligomer laminin 211. There are currently no treatments for MDC1A, and there is an incomplete understanding of disease pathogenesis. Zebrafish, due to their high degree of genetic conservation with humans, large clutch sizes, rapid development, and optical clarity, have emerged as an excellent model system for studying rare Mendelian diseases. They are particularly suitable as a model for muscular dystrophy because they contain at least one orthologue to all major human MD genes, have muscle that is similar to human muscle in structure and function, and manifest obvious and easily measured MD related phenotypes. In this review article, we present the existing zebrafish models of MDC1A, and discuss their contribution to the understanding of MDC1A pathomechanisms and therapy development.

## Introduction

LAMA2-related congenital muscular dystrophy (CMD; LAMA2-MD), also called merosin deficient CMD or MDC1A, is the most common subtype of CMD (Schorling et al., 2017; Sframeli et al., 2017; Mohassel et al., 2018; Mercuri et al., 2019). MDC1A is an autosomal recessive neuromuscular disorder caused by mutations in laminin α2 (*LAMA2*, Helbling-Leclerc et al., 1995; Holmberg and Durbeej, 2013). Complete loss of LAMA2 protein leads to an early onset clinical phenotype featuring severe, diffuse muscle weakness and wasting, demyelinating peripheral neuropathy, and pauci-clinical central nervous system abnormalities, including white matter changes and, in some cases, structural brain lesions (Quijano-Roy et al., 1993; Menezes et al., 2014; Oliveira et al., 2018). The disease is associated with significant co-morbidities, including wheelchair dependence and respiratory failure, and early mortality in some cases (Dimova and Kremensky, 2018). A less common entity is partial merosin deficiency, a disorder caused by partial loss of LAMA2 expression/function that is associated with a later onset, milder form of muscular dystrophy (Nguyen et al., 2019). Both MDC1A and partial merosin deficiency, as well as other rare clinical phenotypes associated with *LAMA2* mutations, are all classified as LAMA2-MD (Oliveira et al., 2018; Verma et al., 2018; Amin et al., 2019). Currently, there are no treatments for LAMA2-MD, and there is an incomplete understanding of disease pathogenesis.

### Laminins

Laminins are high molecular weight glycoproteins expressed abundantly in the basal lamina, a specialized layer of the extracellular matrix (ECM; Aumailley, 2013). Laminins are multidomain heterotrimeric proteins comprised of α, β and γ polypeptide chains (Mohassel et al., 2018), which come in five (LAMA1–5), four (LAMB1–4) and three (LAMC1–3) genetic variants, respectively (Aumailley, 2013; Yurchenco et al., 2018). The polypeptide chains fold in a similar cross-shaped pattern, with distinct structural domains performing specific functions, such as facilitation of self-assembly of most laminins into large polymers by the globular laminin N-terminal (LN) domain (Hohenester, 2019). A few of the polypeptide chains, such as a4 lack the LN domain and therefore do not self-assemble (Aumailley, 2013). Based on the chain composition, more than 15 laminins have been identified in humans (Colognato and Yurchenco, 2000; Sztal et al., 2011). In zebrafish, 12 laminin-encoding genes have been found, out of which 10 have mammalian orthologs, with evolutionarily conserved function (Sztal et al., 2011), whereas two of them, *lamb1b* and *lamb2l*, have no mammalian orthologs (Sztal et al., 2011). Two human laminin-encoding genes, *LAMB3* and *LAMC2*, have not been found in zebrafish (Sztal et al., 2011). Laminins play essential roles in many tissues and organs during development (Yao, 2017). In zebrafish, laminins are involved in myriad developmental processes spanning multiple organ systems (Table 1).

**Table 1:** Examples of developmental processes where laminins are involved.

Developmental process	Genes	References
Neuronal migration	*lama1*	Sittaramane et al. (2009)
Brain morphogenesis	*lamb1*, *lamc1*	Gutzman et al. (2008)
Axon-axon interactions, axon guidance	*lama1*	Paulus and Halloran (2006) and Wolman et al. (2008)
Notochord and blood vessel formation	*lama1*, *lamb1, lamc1*	Parsons et al. (2002) and Pollard et al. (2006)
Establishment of liver and pancreas left-right asymmetry	*lamb1a*	Hochgreb-Hägele et al. (2013)
Fin development	*lama5*	Webb et al. (2007)
Myocardial function	*lama4*	Knöll et al. (2007)
Retinal differentiation and maintenance	*lama1*, *lamb1, lamc1*	Biehlmaier et al. (2007)
Eye development	*lama1*, *lamb1, lamc1*	Semina et al. (2006); Zinkevich et al., 2006 and Lee and Gross (2007)

Several laminin genes are expressed during skeletal muscle development in zebrafish. Some of these, like *lama2*, *lama4*, *lamb2*, and *lamc1*, are detected as early as 24 hpf (when myogenesis begins) and persist in the post-juvenile stages, whereas others (*lamb1*, *lamb4*, and *lamc3*) have only a brief-expression during early muscle development (Sztal et al., 2011). For example, during zebrafish early skeletal muscle development, lamb1 and lamc1 are required for fast muscle fiber elongation, orientation, and their attachment at the myotendinous junctions (MTJs), the primary site of force transmission (Snow et al., 2008; Snow and Henry, 2009). Zebrafish* lamb1* and *lamc1* mutants and morphants show delayed or impaired muscle fiber elongation, non-parallel orientation of fibers in the myotome, and defects in MTJ morphogenesis (Snow et al., 2008). Lama4 is essential for mechanical stability in zebrafish skeletal muscle (Postel et al., 2008). In *lama4* morphants, recruitment of focal adhesion proteins integrin-linked kinase (ilk) and paxillin at the MTJs is impaired, resulting in detachment of myofibers and their surrounding sarcolemma from the MTJ complex (Postel et al., 2008). Lama2 was also shown to be important for zebrafish muscle development and relevant studies on its role in this process are discussed below.

### Laminin α2

The laminin α2 (*LAMA2*) gene encodes the alpha2 chain and constitutes a subunit of several laminin proteins (Tunggal et al., 2000; Aumailley et al., 2005): Laminin 211 (Laminin α2β1γ1, Laminin 2 or merosin; Durbeej, 2015; Barraza-Flores et al., 2020), Laminin α2β2γ1 (Laminin 221, Laminin 4 or S-merosin; Patton et al., 1997) and Laminin 213 (Laminin α2β1γ3, Laminin 12; Koch et al., 1999; Ido et al., 2008). LAMA2 is the major laminin isoform expressed in the vertebrate muscle system (Sztal et al., 2012).

The zebrafish *lama2* gene, representing the ortholog of human *LAMA2*, maps to chromosome 20 and is expressed in the nervous system, head, otic vesicle, adaxial cells, and skeletal muscle (Sztal et al., 2011). Mutations in zebrafish *lama2* results in a type of muscular dystrophy phenotypically similar to the human MDC1A (Hall et al., 2007), which identifies zebrafish as a suitable model for understanding this disease and for development of therapies.

## Zebrafish Models of Muscular Dystrophies

Studies of animal models of muscular dystrophies have proven essential for a better understanding of the pathogenesis of these disorders and for developing disease-specific therapies (Saunier et al., 2016; Widrick et al., 2019). Research using mouse models for LAMA2-MD have identified potential therapeutic strategies, which, in turn, have led to improvements in murine disease pathology and survival (Miyagoe-Suzuki et al., 2000; Meinen et al., 2007, 2011; Vishnudas and Miller, 2009; McKee et al., 2017; Reinhard et al., 2017; Willmann et al., 2017; Mohassel et al., 2018; Yurchenco et al., 2018).

**Figure 1:**
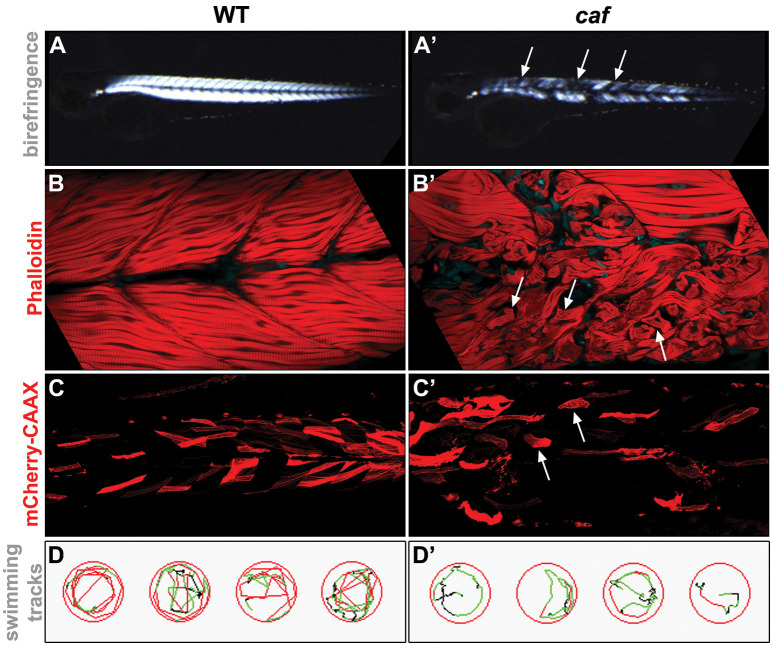
Examples of experimental approaches used for the phenotypical analysis of the zebrafish LAMA2-related congenital muscular dystrophy (CMD, LAMA2-MD) model. **(A,A’)** Birefringence assay. The organization of muscle fibers can be seen by polarized light. Detached muscle fibers in the *caf* mutant show up as dark regions in the muscle (arrows). **(B,B’)** Whole-mount staining. Phalloidin stains the actin filaments in the muscle. Muscle fibers detached from the myotendinous junctions (MTJs) in the *caf* mutant can be easily identified (arrows). **(C,C’)** Injected fluorescent marker. *unc53*:mCherry-CAAX-pA construct (Zhao et al., 2019), which marks muscle cells, was injected into 1-cell stage embryos and visualized by live imaging at 3 dpf. Detached fibers in the *caf* mutant can be easily identified (arrows). **(D,D’)** Swimming assay. Swim behavior can be tracked and quantified using Viewpoint Zebrabox software (Viewpoint Life Sciences Inc.). Time spent moving, distance traveled and speed of movement are useful indicators of muscle function. Fewer tracks and lower speed (indicated by green tracks) are seen in the recorded* caf* mutants.

Recently, zebrafish have emerged as an excellent animal model for studying human muscle diseases, mainly due to their highly similar skeletal muscle, with conserved genetic, molecular and histological features (Telfer et al., 2010; Berger and Currie, 2012; Gibbs et al., 2013). Also, external fertilization, a large number of offspring, rapid embryonic development, optical transparency of embryos and larvae, combined with the ability to easily absorb pharmacological compounds, make zebrafish an excellent tool for studying disease pathomechanisms and identifying potential therapeutic targets (Zon and Peterson, 2005; Gibbs et al., 2013; Waugh et al., 2014; MacRae and Peterson, 2015; Cassar et al., 2020; Fazio et al., 2020). Importantly, readily available and easily applied experimental approaches allow for efficient and rapid assessment of structural and functional damage of the muscular system in the numerous zebrafish dystrophy models. For example, comprehensive phenotypic analysis of muscle damage can be easily done by using birefringence assay (Figures 1A,A′; Berger et al., 2012), histochemistry or immunohistochemistry staining (Figures 1B,B′), injections with fluorescently-tagged markers (Figures 1C,C′; Lombardo et al., 2012) or vital dyes, such as Evans Blue Dye (EBD; Smith et al., 2015) followed by live imaging, swimming assay to assess motor behavior (Figures 1D,D′; Zon and Peterson, 2005; Gibbs et al., 2013; Smith et al., 2013), and other equally useful techniques (Gibbs et al., 2013; MacRae and Peterson, 2015).

Zebrafish models have been developed for many human diseases, such as glioblastoma (Gamble et al., 2018), eye diseases (Moosajee et al., 2008; Bryan et al., 2016), cardiovascular disorders (reviewed in Gut et al., 2017), and kidney diseases (reviewed in Jerman and Sun, 2017), to name a few. Various human muscle disorders, such as Duchenne muscular dystrophy (DMD; Bassett and Currie, 2003; Bassett et al., 2003; Widrick et al., 2016), Laminin α2-associated muscular dystrophy (Jacoby et al., 2009), Ullrich CMD (Telfer et al., 2010), dystroglycanopathies (reviewed in Hewitt, 2009; Lin et al., 2011; Bailey et al., 2019), facioscapulohumeral muscular dystrophy (Mitsuhashi et al., 2013; Pakula et al., 2019), X-linked myotubular myopathy (Dowling et al., 2009; Lawlor et al., 2016; Sabha et al., 2016), and nemaline myopathies (Telfer et al., 2012; Sztal et al., 2015), also have been modeled in zebrafish (reviewed in Nance et al., 2012; Gibbs et al., 2013; Lek et al., 2015; Li et al., 2017).

## Zebrafish Models for Lama2-MD

To date, only a handful of zebrafish models for LAMA2-MD have been developed, even though there are more than three hundred *LAMA2* gene variants associated with human disease (Oliveira et al., 2018).

The first zebrafish model of LAMA2-MD was described by *Currie and colleagues* (Hall et al., 2007) and was identified through complementation studies between dystrophic mutants generated through an N-ethyl-N-nitrosourea (ENU) mutagenesis screen at the University of Tubingen, Germany (Granato et al., 1996). Homozygous mutant zebrafish carrying either *teg15a* or *tk209* recessive mutant allele, show impaired swimming behavior, severe muscle loss, and detached myofibers. Based on the specific shape of the detached fibers, which resemble cotton candy, they named this mutant *candyfloss* (*caf*). The two caf alleles, *caf^teg15^* and *caf^tk209^*, represent loss-of-function mutations in *lama2* gene, and both homozygous mutants exhibit a loss of lama2 protein expression, with similar degenerative muscle phenotype, death by 16 dpf in the majority of cases, and lack of progeny for the small percent of surviving mutants. The mutations have been mapped to the globular domain of *lama2*, which is required for binding to dystroglycan (Hall et al., 2007), a component of the dystrophin-associated glycoprotein complex (DGC) involved in attaching the muscle fibers to the ECM (Sztal et al., 2012). *caf* mutations are located within amino acid residues conserved in humans and mice where known human LAMA2-MD mutations have been identified (Hall et al., 2007).

Using a birefringence assay as a screening tool, it was shown that the muscle defects present in the *caf* zebrafish resemble those described in human patients with LAMA2-MD, namely a stochastic pattern of myofiber detachment from the MTJs. This detachment affects both slow and fast muscle fibers (Sztal et al., 2012), is muscle cell-autonomous, and is dependent on the motor activity of the muscle (Hall et al., 2007; Thomasi et al., 2018). Notably, even though the detachment of the damaged fibers happens rapidly, they maintain the integrity of their sarcolemma, in contrast to what is happening in the muscle of the DMD zebrafish model *sapje* (Bassett et al., 2003; Smith et al., 2015). This is well demonstrated through the use of EBD injections, with *caf* zebrafish showing limited uptake into the sarcoplasm (Hall et al., 2007; Smith et al., 2015). A similar finding of limited/reduced EBD uptake is observed in the *dy* mouse model of LAMA2-MD (Straub et al., 1997), suggesting that impairment of membrane integrity is less prominent in LAMA2-MD vs. other MDs, and also supporting the validity of the muscle phenotype of *caf* zebrafish.

Of note, through elegant *in vivo* time-lapse experiments using various fluorescently-tagged constructs, the specific properties of the *lama2*-deficient myofibers were characterized in detail (Hall et al., 2019). The authors showed these fibers are long-lived, and undergo extensive cellular remodeling by extending protrusions to re-attach to the ECM. They display the formation of new pre-myofibers and undergo nuclear fusion with nearby satellite cells, all processes that indicate that repair, regeneration, and survival mechanisms are activated in the* lama2*-deficient myofibers. Importantly, the authors showed this is not the case in dystrophin-deficient fibers (Hall et al., 2019).

More recently, another zebrafish model for LAMA2-MD has been characterized (Gupta et al., 2012; Smith et al., 2017). The *lama2^cl501^* mutant, also identified through an ENU mutagenesis screen (Gupta et al., 2011), carries a mutation in a highly conserved splice site located in the coiled-coil α-helical domain in the long arm of *lama2*, which is required for binding of LAMA2 to the other laminins in the heterotrimeric complex. This mutation results in a complete loss-of-function due to defective splicing of the* lama2* mRNA. The phenotype of *lama2^cl501^* mutants is essentially identical to that of *caf* zebrafish, with early-onset muscle degeneration due to detachment of fibers from the MTJs and death by 15 dpf (Gupta et al., 2012). Importantly, the detachment of the myofibers from the MTJs in *lama2^cl501^* happens without the loss of sarcolemmal integrity, similar to *caf* mutants. These mutants show reduced laminin expression in the basal membrane at the MTJs complexes, smaller myotomes indicative of growth defects, disorganized sarcomere structure, and increased number of necrotic fibers. Also, *lama2^cl501^* mutants exhibit brain and eye defects (Gupta et al., 2012). Pathogenesis of *lama2^cl501^* is similar to that of human patients with MDC1A, making this mutant another excellent animal model for identifying potential therapies for MDC1A.

Additional research looking at genetic interactions between *lama2* and other dystrophic genes has contributed to our understanding of the specific pathomechanism(s) by which the muscle damage occurs in LAMA2-MD (Sztal et al., 2012). LAMA2, as the major muscle isoform, regulates attachment of myofibers to the ECM either through the dystroglycan complex or through integrin pathways (Tunggal et al., 2000; Pozzi et al., 2017). However, proteins such as dystroglycan (Ervasti and Campbell, 1993; Lisi and Cohn, 2007), dystrophin (Bassett and Currie, 2004), integrin-α7 (Postel et al., 2008), or ilk (Postel et al., 2008) that interact directly or indirectly with LAMA2, play important roles in modifying the LAMA2-MD phenotype. Systematic epistatic experiments in this study (Sztal et al., 2012) showed that concomitant loss of *ilk and dmd* (dystrophin), or *ilk* and *DAG1* (dystroglycan) result in a more severe dystrophic phenotype than the loss of *lama2* or either one alone. Also, the authors show that the phenotype of *lama2/ilk*, *lama2/dmd*, or *lama2/dag1* double homozygous mutants is less severe than the one exhibited by the *ilk/dmd* or *ilk/dag1* mutants, implicating other laminins, in addition to *lama2*, in maintaining the attachment of myofibers to the ECM. Further, by injecting either *lama4* or *lama1* morpholino in *lama2* mutants, Sztal et al. (2012) showed that *lama1*, but not *lama4*, also plays a significant role in this process.

A key outcome of the studies using the *caf* and *lama2^cl501^* zebrafish models was the ability to discriminate between this disorder and DMD, another severe form of muscular dystrophy (Bassett et al., 2003; Bassett and Currie, 2004; Widrick et al., 2016). In muscle from the DMD zebrafish model *sapje* the detached fibers show significant sarcolemmal damage, followed by rapid and increased apoptosis and/or necrosis (Bassett et al., 2003), whereas muscle from the *caf* and *lama2^cl501^* zebrafish fully detaches without concomitant sarcolemmal damage (Hall et al., 2007; Gupta et al., 2012). Also, detached myofibers in *lama2* zebrafish show increased survival and regeneration due to the up-regulation of *lama4* in detached fibers (Sztal et al., 2012).

## Therapeutic Strategies for LAMA2-MD—Lessons from Zebrafish

Several studies using *lama2* zebrafish models identified potential therapeutic strategies for LAMA2-MD (Sztal et al., 2012; Smith et al., 2017; Hall et al., 2019). Results from studies in other dystrophic zebrafish models can also be translated and applied to LAMA2-MD (Goody et al., 2012; Kawahara et al., 2014; Widrick et al., 2019; Wood et al., 2019).

### Drug Screening and Drug Therapy

Studies by Smith et al. (2017) identified and characterized a very early coiling defect in the *lama2^cl501^* fish, which can be used as a measurable and reliable phenotype for drug screening. The mutant fish complete significantly fewer tail coiling movements compared to their wild type siblings (Smith et al., 2017). Importantly, this phenotype manifests only in *caf* and *lama2^cl501^*fish, not in DMD mutants. This early phenotype is consistent with the early perinatal changes observed in LAMA2-MD mouse models (Mehuron et al., 2014), and mirrors the congenital onset phenotype of patients. Therefore, this zebrafish model may be effectively used to identify drug therapies that act at early stages in the LAMA2-MD disease process, which then could be translated into mouse models and clinical trials.

Recent studies using an integrin beta1 zebrafish (*itgβ1*) showed that targeting LAMA2 binding partners, such as integrin, could also provide insights into putative drug therapies for LAMA2-MD (Wood et al., 2019). *itgβ1*-deficient fish displayed increased amounts of LAMA2 and collagen at the ECM, indicating that inhibition of itgβ1 in *lama2*-deficient models might ameliorate the LAMA2-MD phenotype. Injections of the peptide RGD, an itgβ1 inhibitor, led to increased myofiber stability at the basal lamina in *caf* zebrafish, by increasing the levels of lama2 at the ECM (Wood et al., 2019).

Additional insights into using zebrafish models for the development of drug therapies for muscular dystrophies were provided by studies modulating nicotinamide adenine dinucleotide (NAD+) biosynthesis in *dag1* and* itga7* dystrophic morphants (Goody et al., 2012). NAD+ synthesis, mediated by the muscle-specific nicotinamide riboside kinase 2b (nrk2b), was shown to be essential for lamc1 polymerization at the MTJs and identified additional regulators of muscle morphogenesis in the cell adhesion signaling pathway (Goody et al., 2010). Exogenous supplementation of NAD+ or overexpression of its downstream effector, paxillin, ameliorate the dystrophic phenotype, by increasing the MTJ-basement membrane organization through laminin augmentation (Goody et al., 2012).

### Gene Therapy and Protein Replacement Therapy

Recent studies in the *caf^teg15^* zebrafish model showed that expressing *lama2* or injecting lama2 rescues the LAMA2-MD dystrophic fiber phenotype (Hall et al., 2019). Generalized expression of *lama2* under a heat-shock promoter during embryonic development or muscle-specific overexpression of *lama2* in *caf* fish led to normal levels and correct distribution of laminin at the MTJs and complete rescue of the dystrophic phenotype (Hall et al., 2019). Driving the expression of *lama2* later in development, after the dystrophic phenotype is fully established, resulted in a significant decrease in the number of detached fibers, increased survival, remodeling, repair and reattachment of detached fibers (Hall et al., 2019). Intramuscular delivery of Laminin111, a laminin complex similar to Laminin211 shown to functionally replace Laminin211 in an MCD1A mouse model (Van Ry et al., 2014), increased the population of muscle stem cells and resulted in significant improvement of the *caf* phenotype (Hall et al., 2019). This is similar to what has been described for laminin replacement therapy in DMD and *alpha7 integrin*-null mouse models (Rooney et al., 2009a,b; Goudenege et al., 2010) and provides additional validation of the therapy, as well as of the model as a vehicle for discovery and development of therapies.

### Caveats of Using Zebrafish as the LAMA2-MD Disease Model

The above studies describing LAMA2-MD zebrafish models, together with the increasing number of studies modeling other human diseases in zebrafish (Steffen et al., 2007; Wood and Currie, 2014), prove the amenability of zebrafish as an organism for advancing our understanding of pathogenic mechanisms and therapies development. However, we should mention that a few caveats should be taken into consideration when translating the results from the LAMA2-MD zebrafish to human patients with LAMA2-MD.

LAMA2-MD pathophysiology shows slight differences between human patients and zebrafish models. For example, in humans, the LAMA2-MD dystrophic phenotype is associated, besides other features, with increased atrophy and apoptosis, defective regeneration and repair, depletion of satellite cell pools, upregulated autophagy and abnormal proteasome-dependent degradation (Durbeej, 2015). These changes have yet to be thoroughly examined in zebrafish models of LAMA2-MD. Also, evaluating non-muscle phenotypes associated with LAMA2 mutations presents challenges in the zebrafish. Importantly, myelination is distinctly different in zebrafish compared to mammals, with peripheral myelin expressing myelin basic protein and not MPZ or PMP22. Thus, studying mechanisms related to white matter disease and peripheral neuropathy may not be feasible in zebrafish, and addressing the impact of therapeutic interventions on these features of disease not possible.

Designing drug screens for LAMA2-MD in zebrafish requires taking into consideration that detachment of myofibers in *caf* mutants is movement- and mechanical load-dependent (Hall et al., 2007). Therefore, it is necessary to ensure the drugs tested do not affect swimming behavior. Fish immobilized due to highly toxic drugs, for example, would lead to the identification of false-positive drug hits.

Overall, there is therefore the need to balance the advantage of the zebrafish with these shortcomings. The integration of observations in *caf* mutants with other *in vivo* models of LAMA2-MD should greatly aid in their translatability. In particular, several mutant mouse models accurately phenocopy key aspects of the human disease (Gawlik and Durbeej, 2020), and provide an opportunity to test and validate findings from the *caf* mutants and to determine their relevance to non-muscle systems. This is particularly true concerning therapy development, where a pipeline of large scale drug screening in zebrafish combined with testing and validation in the mouse may yield candidate therapeutics with the highest potential for successful translation to patients. The establishment of a similar pipeline crossing multiple species was recently reported for congenital muscle disease due to *RYR1* mutation (Volpatti et al., 2020).

## Conclusions

Zebrafish models of human diseases contribute significantly to our understanding of underlying pathogenic mechanisms, characterization of signaling pathways regulating them, and development of therapeutic strategies. The main strengths of the zebrafish model are a large number of offspring, rapid embryonic development, and optical transparency of the embryos, which allow for successful screening approaches, from drug discovery to genome-scale CRISPR and genetic modifiers screening (reviewed in Gut et al., 2017).

Zebrafish is an excellent model organism to study LAMA2-MD, as they mirror the genetics and motor phenotypes of patients and carry important advantages for pathway analyses and drug discovery. Zebrafish are extremely amenable to high-throughput chemical screening to identify therapeutic drugs for LAMA2-MD (MacRae and Peterson, 2015). This approach has been successfully used in other zebrafish models of human disease (Bootorabi et al., 2017; Jardine et al., 2020; Gut et al., 2017; Matsuda et al., 2018). Furthermore, genome-editing technologies such as TALENs and CRISPR/Cas systems are easily applied to zebrafish and could be used to generate and study a large number of patient-specific mutations (Zhang et al., 2018; Giardoglou and Beis, 2019; Lek et al., 2020). Despite some limitations (Gut et al., 2017), these genome-editing approaches allow for the generation of a theoretically unlimited number of zebrafish mutants, which could ultimately enable scientists to systematically and comprehensively study full allele series for disorders such as LAMA2-MD. Lastly, performing genetic modifiers screens in *caf* zebrafish with methodologies including ENU mutagenesis and CRISPR gene editing (McGovern et al., 2015; Quattrocelli et al., 2017a,b; Rahit and Tarailo-Graovac, 2020; Volpatti et al., 2020) should enable the identification of genetic interactions and novel disease modifiers, data which would greatly advance our understanding of the pathomechanisms and phenotypic variability of LAMA2-MD.

## Author Contributions

JD: conception and final approval. JD and LF: design and critical revisions. LF: drafting the article.

## Conflict of Interest

The authors declare that the research was conducted in the absence of any commercial or financial relationships that could be construed as a potential conflict of interest.
